# The altered fate of aging satellite cells is determined by signaling and epigenetic changes

**DOI:** 10.3389/fgene.2015.00059

**Published:** 2015-02-20

**Authors:** Maura H. Parker

**Affiliations:** Clinical Research Division, Fred Hutchinson Cancer Research Center, Seattle, WA, USA

**Keywords:** skeletal muscle, satellite cells, niche, aging, quiescence, senescence

## Abstract

Skeletal muscle is a striated tissue composed of multinucleated fibers that contract under the control of the somatic nervous system to direct movement. The stem cells of skeletal muscle, known as satellite cells, are responsible for muscle fiber growth, turnover, and regeneration. Satellite cells are activated and proliferate in response to stimuli, and simplistically, have two main fates—to repopulate the satellite cell niche, or differentiate to regenerate or repair muscle fibers. However, the ability to regenerate muscle and replace lost myofibers declines with age. This loss of function may be a result of extrinsic changes in the niche, such as alterations in signaling or modifications to the extracellular matrix. However, intrinsic epigenetic changes within satellite cells may also affect cell fate and cause a decline in regenerative capacity. This review will describe the mechanisms that regulate cell fate decisions in adult skeletal muscle, and how changes during aging affect muscle fiber turnover and regeneration.

## AGING, SARCOPENIA, AND THE SATELLITE CELL

Aging is characterized by a time-dependent accumulation of cellular damage. Cells suffer damage as a result of chronological aging, as well as replicative aging, which occurs as cells proliferate in response to various stimuli. Stem cells are thought to be protected from the effects of aging by quiescence, a state of cellular hibernation which provides cushioning from the stresses associated with cell proliferation, thereby limiting cellular damage. However, quiescent cells are not dormant. Instead, quiescent stem cells actively maintain their state; they are poised for action, waiting for signals that activate proliferation. Aging is associated with the inability to maintain stem cell quiescence, which increases the chances of stem cell damage, and results in a loss of stem cell self-renewal and regenerative capacity.

Aging is also associated with a gain of cellular senescence. Senescent cells are alive and metabolically active, but have lost the ability to divide. The primary purpose of senescence is to prevent propagation of damaged cells. Senescent cells are resistant to apoptosis, and are normally cleared by the immune system. Once cleared, tissue-specific repair mechanisms are activated and lost cells are replaced. During aging, senescent cells accumulate, which increases tissue inflammation, as senescent cells secrete pro-inflammatory cytokines, such as TNFα and IL-6. Thus, an increase in senescence, combined with a decrease in regenerative capacity, is predicted to result in a net loss of cells and/or tissue.

In skeletal muscle, aging is manifested as sarcopenia, the gradual loss of muscle mass and function in the absence of an attributable disease. Skeletal muscle is eventually replaced by fatty and fibrous tissue, which results in functional impairment of the muscle and physical disability. In the United States, sarcopenia occurs in approximately 45% of the population over the age of 60, and upward of 50% of muscle fibers are lost from limb muscles by the age of 80 (Janssen et al., 2004; Faulkner et al., 2007). However, sarcopenia can occur at any age as a result of disuse or malnutrition. In younger individuals, the loss of muscle mass is reversible, whereas in older or geriatric individuals, muscle loss appears to be irrecoverable.

The ability to generate skeletal muscle during post-natal growth and to regenerate skeletal muscle in adults is almost exclusively due to the action of Pax7-expressing satellite cells, the stem cells of skeletal muscle (Lepper et al., 2011; Murphy et al., 2011; Sambasivan et al., 2011). During the process of muscle regeneration, satellite cells are activated and proliferate, and adopt one of two cell fates: differentiation to generate or repair muscle fibers, or a return to quiescence to repopulate the satellite cell niche.

Given the role of satellite cells in post-natal muscle growth and adult muscle regeneration, it is reasonable to hypothesize that satellite cells are responsible for maintaining muscle mass and myonuclei number through aging. If true, then sarcopenia is predicted to be the result of a loss of satellite cell number, or a failure of satellite cells to function in aged individuals. Indeed, many studies have shown an age-related decline in the number of satellite cells and/or an age-related loss of satellite cell function (Roth et al., 2000; Conboy et al., 2003; Shefer et al., 2006; Day et al., 2010; Chakkalakal et al., 2012; Sousa-Victor et al., 2014). Moreover, transplantation of young satellite cells into the muscle of progeroid mice extends lifespan and ameliorates degenerative changes in skeletal muscle (Lavasani et al., 2012).

A more recent study challenges the notion that loss of satellite cells or satellite cell function is responsible for age-related sarcopenia (Fry et al., 2015). In this study, young mice were treated briefly with tamoxifen to deplete satellite cells by Pax7-dependent activation of diphtheria toxin A (DTA) expression, and then allowed to age naturally. The results clearly show that satellite cell depletion does not accelerate age-related sarcopenia, but does affect the ability of skeletal muscle to respond to acute injury. This study highlights the need to better understand myonuclear turnover in adult muscle, and raises the question of whether satellite cells play a role in adult muscle maintenance.

A small population of satellite cells remains after tamoxifen treatment, and appears to increase in number over time in the gastrocnemius, tibialis anterior, and extensor digitorum longus muscles. Radiation-induced depletion of functional satellite cells in mice also results in survival of a small sub-population of satellite cells, which maintain the ability to contribute to repair and regeneration of skeletal muscle (Heslop et al., 2000). Moreover, transplant of a very small number of satellite cells, associated with a single muscle fiber, has the ability to generate a significant number of donor-derived muscle fibers, and contribute to the recipient satellite cell niche (Collins et al., 2005). Therefore, it is possible that the small number of satellite cells that remain in tamoxifen-treated mice provides enough cells to maintain muscle mass until other age-related changes occur, at which point loss of satellite cell function manifests as sarcopenia.

Age-related changes can be satellite cell-intrinsic or extrinsic. Satellite cells exist within a niche that consists of surrounding cells and the extracellular matrix (ECM), which provide biochemical and biophysical signals that direct regeneration and self-renewal. Age-related changes to the niche have the potential to affect satellite cell fate by altering environmental cues, resulting in aged satellite cells failing to re-enter quiescence, aberrantly entering senescence and/or failing to prevent fibrosis.

Satellite cells from aged and young muscle appear to proliferate *ex vivo* with similar rates (George et al., 2010; Alsharidah et al., 2013; Verdijk et al., 2014). The inability of aged satellite cells to show the effects of aging in a culture dish suggests that the aged muscle environment is to blame for the decline in regenerative capacity. However, studies with human cells suggest that culturing with 20% fetal calf serum masks differences between young and aged satellite cells, and demonstrate that culturing with human sera of the same age reveals a delayed response to activating stimuli and reduced proliferation (Barberi et al., 2013). Moreover, reduced regeneration in adult mice transplanted with FACS sorted geriatric satellite cells as compared to adult mice transplanted with adult satellite cells suggests a cell-intrinsic change that affects aged satellite cell function (Sousa-Victor et al., 2014).

Together, these data this suggests that satellite cell-intrinsic changes, combined with satellite cell-extrinsic changes within the niche alter cell fate decisions, and manifest as inefficient skeletal muscle repair, resulting in sarcopenia. This review will examine how satellite cell-extrinsic and satellite cell-intrinsic changes during aging affect satellite cell fate decisions and implicate the loss of satellite cell function as causative in sarcopenia.

## AGE-RELATED FIBROSIS AND SATELLITE CELL FATE

During the later stages of normal regeneration, a sub-population of macrophages in the muscle secrete TGFβ, which directs muscle-resident fibroblasts to secrete ECM proteins that reconstitute the basal lamina and the reticular lamina that surround muscle fibers. The ECM provides mechanical support and a scaffold to orient the fibers during regeneration (Sanes, 2003). Activation of TGFβ/activin signaling in cells specifically phosphorylates Smad2 and Smad3, stimulating nuclear localization and regulating gene expression. TGFβ-mediated phosphorylation of Smad3 is specifically required for expression of collagen and ECM components in fibroblasts, and for activation and proliferation in satellite cells (Ge et al., 2011, 2012).

During aging, skeletal muscle fibers are progressively replaced by adipose and fibrotic tissue, which is exacerbated by injury (Brack et al., 2007; Paliwal et al., 2012). The formation of excessive connective tissue, also known as fibrosis, is a characteristic feature of sarcopenia. A change in intensity and duration of the macrophage response in aged skeletal muscle results in a higher level of TGFβ signaling in skeletal muscle (Zacks and Sheff, 1982; Carlson et al., 2008). This extends the phase of protein deposition by skeletal muscle fibroblasts, resulting in an increased level of ECM proteins and the presence of atypical types of collagen (Marshall et al., 1989; Alexakis et al., 2007). Moreover, less collagen turnover and more collagen cross-linking results in a densely packed lamina that increases muscle stiffness and potentially limits skeletal muscle function.

Increased TGFβ signaling inhibits satellite cell activation and proliferation (Allen and Boxhorn, 1987, 1989; Rathbone et al., 2011). Sustained TGFβ signaling in aged muscle is expected to decrease satellite cell proliferation, stimulate proliferation of fibroblasts in skeletal muscle, and increase expression of ECM proteins. Specifically, loss of satellite cell-derived signaling to muscle-resident fibroblasts relieves repression of collagen Ia1, collagen IIIa1, collagen VIia2, and fibronectin expression (Fry et al., 2014). Therefore, satellite cells, in addition to participating in the generation and repair of muscle fibers, are also responsible regulating ECM production and preventing fibrosis.

High levels of Wnt3a induce skeletal muscle fibrosis in mice, suggesting there may be a link between TGFβ and Wnt signaling in promoting fibrosis in aged muscle (Brack et al., 2007). Indeed, aged mice display an increase in the level of a serum factor that promotes Wnt activity, and this serum factor is postulated to promote excessive production of ECM proteins. This serum factor may be the complement protein, C1q, which can bind Fzd receptors and activate canonical Wnt signaling (Naito et al., 2012; Watanabe et al., 2014).

One study suggests that Wnt3a signaling stimulates canonical Wnt signaling and induces a change in cell fate, such that myogenic satellite cells are converted to the fibrogenic lineage (Brack et al., 2007). However, a separate study indicates that injection of a high level of Wnt3a into mouse skeletal muscle stimulates proliferation of a stromal cell population that produces collagen, resulting in replacement of adult skeletal muscle with fibrous tissue (Trensz et al., 2010). Importantly, both age- and disease-related fibrosis can be resolved by injection of DKK1, a Wnt signaling antagonist (Brack et al., 2007; Trensz et al., 2010).

The increase in fibrosis affects the ability of skeletal muscle to function. However, fibrosis also exacerbates the loss of satellite cell function by preventing satellite cell proliferation and self-renewal. Laminin, a primary protein component of the ECM, specifically interacts with integrin receptors on the surface of satellite cells. Satellite cell proliferation depends on a properly organized network of laminin within the basal lamina of the ECM (Ross et al., 2012). Moreover, the laminin-integrin interaction induces cell-intrinsic polarity, which is essential for asymmetric cell division and satellite cell self-renewal (Kuang et al., 2007; Goulas et al., 2012). Therefore, persistent TGFβ and Wnt signaling during aging leads to accumulation of skeletal muscle fibrosis, which disrupts basal lamina architecture, and reduces satellite cell proliferation and self-renewal.

## AGE-RELATED LOSS OF SATELLITE CELL SELF-RENEWAL

### SIGNALING AND SATELLITE CELL SELF-RENEWAL

Impaired skeletal muscle regeneration in aged mice is due, in part, to loss of Notch signaling, and can be restored by forced activation of Notch in aged muscle, or parabiosis of aged mice with young mice (Conboy et al., 2003, 2005; Carlson et al., 2009). Similarly, skeletal muscle aging can be simulated in young mice by inhibition of Notch signaling after acute injury (Conboy and Rando, 2002). Satellite cell-specific deletion of RBP-J, the primary mediator of Notch signaling, in adult muscle results in loss of satellite cells and reduced regenerative capacity, as RBP-J-null satellite cells spontaneously enter the cell cycle and immediately progress through differentiation without self-renewing (Bjornson et al., 2012; Mourikis et al., 2012). Therefore, age-related loss of Notch signaling precludes satellite cell self-renewal, manifesting as a loss of satellite cells and impaired regenerative capacity.

Similarly, conditional deletion of RBP-J, in embryonic myogenic progenitors results in an absence of satellite cells in fetal muscle due to premature differentiation (Vasyutina et al., 2007). In a MyoD-null background, the loss of satellite cells is prevented, presumably due to a delay in myogenic differentiation (Brohl et al., 2012). Myogenic progenitor cells in RBP-J-null/MyoD-null mice are unable to home to the satellite cell niche, in part, due to loss of integrin α7 expression, which mediates the interaction between satellite cells and the basal lamina. Therefore, loss of Notch signaling in aging skeletal muscle, combined with alterations to basal lamina architecture as a result of fibrosis, disrupts satellite cell proliferation and self-renewal.

Diminished Notch activity in satellite cells is due, in part, to an age-related decrease in expression of the Notch receptor ligands, Jag1 and Dll1 (Conboy et al., 2003; Carey et al., 2007; Carlson et al., 2009). Reduced levels of Dll1 in mice results in severe muscle hypotrophy as a result of insufficient satellite cell proliferation (Schuster-Gossler et al., 2007). Jag1 is expressed in a subset of activated satellite cells, and generates asymmetry during cell division by activating Notch signaling in an adjacent receptor-expressing cell (Gnocchi et al., 2009). The Jag1 expressing cell expresses Numb, an antagonist of Notch signaling, and is fated to progress through commitment and differentiation; whereas the Numb-negative satellite cell responding to the Jag1 signal displays high Notch activity, and is fated for self-renewal (Conboy and Rando, 2002; Shinin et al., 2006). Loss of Jag1 expression in aging satellite cells prevents asymmetric Notch signaling, and therefore, prevents self-renewal.

Skeletal muscle regeneration has been postulated to be a balance between Notch and Wnt signaling, such that Notch is required for proliferation and self-renewal of satellite cells, and canonical Wnt signaling is required for induction of differentiation (Conboy and Rando, 2002; Conboy et al., 2003; Brack et al., 2007, 2008). However, tamoxifen-mediated deletion of β-catenin specifically in satellite cells suggests that canonical Wnt signaling is not required for differentiation during adult muscle regeneration (Murphy et al., 2014).

Wnts activate canonical and non-canonical pathways. In aging hematopoietic stem cells, a switch from canonical to non-canonical Wnt signaling causes a loss in stem-cell polarity and reduces regenerative potential (Florian et al., 2013). It is intriguing to hypothesize how a shift in which Wnt pathway is activated in aging could affect satellite cell fate and function. Given that non-canonical Wnt signaling in satellite cells specifically stimulates symmetric expansion, an age-related shift in Wnt signaling to the non-canonical pathway may impinge on satellite cell self-renewal, resulting in a loss of regenerative capacity (Le Grand et al., 2009). Therefore, persistent canonical Wnt signaling during aging may prevent satellite cell self-renewal, in addition to stimulating fibrosis.

Self-renewal of satellite cells requires asymmetric cell division and the ability of cells to re-enter and maintain quiescence. Increased expression of MyoD and Myf5 in aged muscle of rats and humans, in the absence of exercise or injury, suggests that satellite cells lose the ability to maintain quiescence during aging (Hameed et al., 2003; Edstrom and Ulfhake, 2005; Raue et al., 2006). Indeed, uninjured muscle from aged mice displays a greater percentage of actively proliferating MyoD-expressing satellite cells, as compared to the muscle of young mice (Chakkalakal et al., 2012). Reduced expression of p27, a cyclin-dependent kinase inhibitor (CDKi), and Sprouty1, a FGF signaling antagonist, is suggested to be the cause of spontaneous release from quiescence in aged satellite cells.

Spry1 expression is restricted to non-cycling satellite cells, and is required for satellite cells to return to and maintain quiescence during regeneration (Shea et al., 2010). Long-term deletion of Spry1 in adult skeletal muscle satellite cells decreases the percentage of label-retaining cells, consistent with the loss of satellite cell quiescence observed in aging. Furthermore, Spry1-null satellite cells are unable to contribute to the generation of myonuclei or the renewal of quiescent satellite cells during skeletal muscle repair. Therefore, loss of Spry1 expression in a subpopulation of aged satellite cells, combined with the increase in FGF2 expression in aged muscle fibers, drives satellite cell depletion by maintaining proliferation and preventing the return to quiescence. This is intriguing, as it suggests that the combination of cell autonomous changes (loss of Spry1 expression) and changes to the niche (increased FGF2 expression) are involved in the loss of satellite cell self-renewal.

The FGF2-induced signal in satellite cells is mediated by many downstream targets, including p38 MAPKs (Cuadrado and Nebreda, 2010). In turn, activated p38 MAPKs phosphorylate a broad range of targets, including MyoD, NF-κB, CREB, and STAT1/3. Conditional deletion of p38α expression, or inhibition p38α activity, promotes Pax7 expression and expansion of satellite cells, and prevents differentiation (Palacios et al., 2010; Brien et al., 2013). Activated and proliferating satellite cells display asymmetric distribution of activated phospho-p38α/β (pp38α/β), such that cells expressing pp38α/β co-express MyoD and progress through myogenic differentiation, whereas, pp38α/β-negative cells revert to quiescence (Jones et al., 2005; Troy et al., 2012).

Aging satellite cells display elevated pp38α/β levels, combined with a loss of asymmetric distribution of pp38α/β, which correlates with a loss of self-renewal (Troy et al., 2012). Partial inhibition of p38α/β activity restores self-renewal of aged satellite cells *in vitro* and reestablishes engraftment potential (Bernet et al., 2014; Cosgrove et al., 2014). Analogously, expression of a ligand-independent constitutively active form of FGFR1 also drives asymmetric localization of pp38α/β in aged satellite cells, and permits satellite cell self-renewal (Bernet et al., 2014).

Like other signaling pathways, the p38α/β pathway does not operate independently. Par-3, an evolutionarily conserved regulator of polarity, colocalizes with pp38α/β in dividing satellite cells (Troy et al., 2012). In asymmetrically dividing radial glia, Par-3 is responsible for asymmetric localization of Mib to the apical daughter cell, which is fated for differentiation (Bultje et al., 2009; Dong et al., 2012). Mib is an ubiquitin ligase that regulates Notch ligand endocytosis in the apical cell, a process that is required for efficient activation of Notch signaling in the basal cell, which is destined for self-renewal. This is strikingly similar to satellite cells, in which high Notch activity marks satellite cells fated for self-renewal.

Therefore, aging disrupts satellite cell self-renewal. Specifically, age-related cell extrinsic changes in expression of signaling ligands, combined with satellite cell-intrinsic alterations in the ability to appropriately respond to signals, disrupt asymmetric cell division and limit satellite cell self-renewal. The inability to self-renew results in a progressive loss of satellite cells, which diminishes competence to respond to acute injury and maintain muscle mass.

### EPIGENETICS AND SATELLITE CELL SELF-RENEWAL

Activation of p38α signaling directs satellite cells toward differentiation, and prevents self-renewal, by repressing expression of Pax7 and Notch1 through localized targeting of Ezh2, a histone methyltransferase, and DNMT3b, a DNA methyltransferase, to each gene (Acharyya et al., 2010; Palacios et al., 2010). Ezh2 is a component of the polycomb repressive complex (PRC2), which in combination with PRC1, establishes and stabilizes repression through post-translational modification of histones.

Post-translation modification of histones is an epigenetic change that marks genes as active or inactive. Methylation of lysine 4 (H3K4me3) of histone H3 is generally associated with active chromatin, while methylation of lysine 27 (H3K27me3) is linked with repressed chromatin (Dilworth and Blais, 2011). A bivalent state can exist in which histone H3 is methylated at both lysine 4 and lysine 27. Notably, the repressive H3K27me3 mark is dominant over the active H3K4me3 mark, and is heritably transmitted to daughter cells (Barski et al., 2007).

Genome-wide analysis of chromatin in young and aged quiescent satellite cells demonstrated that the level of H3K4me3 histone marking was comparable between young and old satellite cells; however, H3K27me3 accumulates and spreads with age in quiescent satellite cells (Liu et al., 2013). Notably, 30% of genes that acquire H3K27me3 were not expressed in either young or old quiescent satellite cells, and less than 0.5% of genes are marked solely by H3K27me3. It is difficult to correlate the global change in H3K27me3 in satellite cells with aging. However, this gain of H3K27me3 marks may reflect a loss of potential, as low levels of H3K27me3 is associated with pluripotency (Mikkelsen et al., 2007; Marks et al., 2012).

The increase in H3K27me3 in aging is thought to be linked to a redistribution of PRC1 and PRC2 complexes. Bivalent domains can be segregated into two types—those that are bound by PRC1 and PRC2, and those that are bound only by PRC2 (Ku et al., 2008). Binding of PRC1 more effectively retains the H3K27me3 mark, thereby maintaining repression. Specifically, PRC1 stabilizes bivalent domains, and reinforces the ability of stem cells and progenitor cells to retain cell fate choices, including self-renewal (Oguro et al., 2010). Therefore, loss of PRC1 is expected to drive satellite cells out of quiescence and prevent satellite cell self-renewal. Indeed, mice lacking Bmi1, a component of the PRC1 complex, show reduced numbers of Pax7^+^Myf5^–^ satellite stem cells, and an increase in Pax7^+^Myf5^+^ and MyoD^+^ committed satellite cells, reminiscent of the age-related loss of quiescence (Robson et al., 2011). Moreover, Bmi1-null mice display a delay in regeneration upon injury. Therefore, cell-extrinsic changes to signaling and cell-intrinsic changes in signal response during aging can produce long-term and heritable results by inducing epigenetic changes.

## AGE-RELATED INDUCTION OF SENESCENCE

Senescent cells are alive and metabolically active, but have lost the ability to divide. Senescence can be induced through several mechanisms, but is most closely associated with aging. The primary purpose of senescence is to prevent propagation of damaged cells. Senescent cells are cleared by the immune system, and lost cells are replaced by tissue-specific repair mechanisms. Age-related changes in the immune system, combined with an increase in the number of senescent cells, may result in the accumulation of senescent cells, which secrete cytokines and other molecules that induce inflammation and inhibit tissue regeneration (Kuilman et al., 2008; Rodier and Campisi, 2011).

Recent studies indicate that satellite cells enter senescence with advanced age. Sousa-Victor et al. (2014) compared adult (5–6 mo), old (20–24 mo), and geriatric (28–32 mo) mice, and showed that old and geriatric mice display a reduced number of satellite cells, but only satellite cells in geriatric mice display a reduced proliferative response. Moreover, skeletal muscle regeneration was marginally reduced in old mice, but is more markedly diminished in geriatric mice. Transplantation of FACS sorted cells from adult, old, and geriatric mice into young mice clearly showed a significant reduction in regenerative potential only in geriatric cells, indicating a cell-intrinsic loss of regenerative capacity with aging.

Mouse and human geriatric satellite cells express p16^INK4A^, and display classic markers of senescence (Sousa-Victor et al., 2014). Silencing of p16^INK4A^ expression in geriatric mouse satellite cells restores regeneration-induced activation of proliferation and reversible quiescence. In parallel experiments, ectopic expression of p16^INK4A^ prevents activation of satellite cells after injury of young muscle. These data suggest that expression of p16^INK4A^ in geriatric satellite cells induces cellular senescence and is responsible for the aging phenotype in skeletal muscle. However, a mild and systemic increase in p16^INK4A^ expression extends longevity in mice, suggesting the dosage of p16^INK4A^ may be important for determining effect (Matheu et al., 2007, 2009).

In young cells, the combined action of PRC1 and PRC2 represses p16^INK4A^ expression through maintenance of H3K27me3 marks, thereby preventing cellular senescence (Jacobs et al., 1999; Bracken et al., 2007; Margueron and Reinberg, 2011). Geriatric satellite cells display an increase in expression of genes normally regulated by PRC1 and PRC2, suggesting that satellite cells may exhibit age-related epigenetic changes. Bmi1, a component of the PRC1 complex, represses expression of p16^INK4A^, and has been suggested to play an important role in delaying aging by preventing cellular senescence (Jacobs et al., 1999). Therefore, if Bmi1 expression or function is lost with aging, satellite cells would be expected to lose the ability to maintain quiescence and eventually senesce.

Notch signaling positively regulates expression of Bmi1, suggesting that loss of Notch signaling in aging satellite cells may reduce Bmi1 expression, de-repress the p16^INK4A^ locus, resulting in satellite cell senescence (Fan et al., 2010; Schaller et al., 2010; Sousa-Victor et al., 2014). Indeed, deletion of Bmi1 in young satellite cells leads to de-repression of the p16^INK4A^ locus, increased expression of p16^INK4A^, which leads to a senescent-like state in young cells and prevents these cells from participating in regeneration (Robson et al., 2011). Moreover, reduced Notch signaling in aged satellite cells allows TGFβ-stimulated phosphorylation of Smad3 to activate expression of CDK inhibitors (CDKis; Beggs et al., 2004; Carlson et al., 2008). Therefore, persistent TGFβ signaling and loss of Notch signaling during skeletal muscle aging increases fibrosis, inhibits satellite cell proliferation, and induces satellite cell senescence.

## CONCLUSIONS AND PERSPECTIVES

Age-related changes within satellite cells and to their niche limit cell fate and function. In the aged niche, satellite cells shift from a poised, quiescent state to the active state in the absence of a regenerative signal. Persistent TGFβ-and Wnt-dependent accumulation of skeletal muscle fibrosis disrupts basal lamina architecture. Dysregulation of Wnt, Notch, FGF, and p38α/β signaling results in a loss of cell polarity, and prevents asymmetric cell division. Age-related loss of Notch activity and persistence of TGFβ activity induce epigenetic changes that de-repress the *CDKN2A* locus and induce expression of p16^INK4A^. These changes combine to drive satellite cells away from normal cell fate decisions—differentiation and self-renewal—toward age-realted senescence. In genome-wide association studies, the p16^INK4A^ locus is genetically linked to the highest number of age-associated pathologies.

Restoring regenerative capacity to aged skeletal muscle could be as simple as replacing aged satellite cells with young satellite cells, and/or modifying signaling pathways to maintain reversible quiescence. However, it appears that the effects of aging culminate in epigenetics and expression of p16^INK4A^. Global demethylation of DNA occurs after fertilization and is required for pluripotency (Guo et al., 2014; Smith et al., 2014). This suggests that skeletal muscle aging may be reversed simply by manipulating the epigenetic memory of satellite cells, and resetting the aging clock to zero.

### Conflict of Interest Statement

The author declares that the research was conducted in the absence of any commercial or financial relationships that could be construed as a potential conflict of interest.
